# DICER1 Syndrome and Cancer Predisposition: From a Rare Pediatric Tumor to Lifetime Risk

**DOI:** 10.3389/fonc.2020.614541

**Published:** 2021-01-21

**Authors:** Anna Maria Caroleo, Maria Antonietta De Ioris, Luigi Boccuto, Iside Alessi, Giada Del Baldo, Antonella Cacchione, Emanuele Agolini, Martina Rinelli, Annalisa Serra, Andrea Carai, Angela Mastronuzzi

**Affiliations:** ^1^ Department of Onco – Hematology and Cell and Gene Therapy, Bambino Gesù Pediatric Hospital (IRCCS), Roma, Italy; ^2^ JC Self Research Institute, Greenwood Genetic Center, Greenwood, SC, United States; ^3^ School of Nursing, College of Behavioral, Social and Health Sciences, Clemson University, Clemson, SC, United States; ^4^ Laboratory of Medical Genetics, Bambino Gesù Children Hospital (IRCCS), Rome, Italy; ^5^ Department of Neuroscience, Bambino Gesù Children Hospital (IRCCS), Rome, Italy

**Keywords:** DICER1, cancer predisposition, pediatric, PPB, cystic nephroma

## Abstract

DICER1 syndrome is a rare genetic condition predisposing to hereditary cancer and caused by variants in the *DICER1* gene. The risk to present a neoplasm before the age of 10 years is 5.3 and 31.5% before the age of 60. *DICER1* variants have been associated with a syndrome involving familial pleuropulmonary blastoma (PPB), a rare malignant tumor of the lung, which occurs primarily in children under the age of 6 years and represents the most common life-threatening manifestation of DICER1 syndrome. Type I, II, III, and Ir (type I regressed) PPB are reported with a 5-year overall survival ranging from 53 to 100% (for type Ir). *DICER1* gene should be screened in all patients with PPB and considered in other tumors mainly in thyroid neoplasms (multinodular goiter, thyroid cancer, adenomas), ovarian tumors (Sertoli-Leydig cell tumor, sarcoma, and gynandroblastoma), and cystic nephroma. A prompt identification of this syndrome is necessary to plan a correct follow-up and screening during lifetime.

## Introduction

DICER1 syndrome is a cancer-predisposing disorder caused by pathogenic variants in the *DICER1* gene (OMIM 606241), which are known to confer a lifetime risks for a variety of neoplastic and dysplastic lesions (1).

Germline *DICER1* variants have been detected in individuals affected with familial pleuropulmonary blastoma (PPB) (2–5), a rare malignant tumor of the lung, which occurs primarily in children under the age of 6 years (6). The International PPB Registry collected data from PPB patients and their families, reporting a variety of tumors in individuals with PPB and/or their relatives (6). A study on 207 carriers of DICER1 pathogenic variants reported that the risk to develop a neoplasm is 5.3% before the age of 10 years and of 31.5% before the age of 60, while in the American general population is estimated to be respectively 0.17 and 6.57% (1, 7). DICER1 syndrome occurs in children and young adults and its clinical presentation may include, beyond PPB, cystic nephroma, ovarian Sertoli-Leydig cell tumor (SLCT), multinodular goiter, cervix embryonal rhabdomyosarcoma, Wilms’ tumor, nasal chondromesenchymal hamartoma, ciliary body medulloepithelioma, differentiated thyroid carcinoma, pituitary blastoma, pineoblastoma, and sarcomas of different sites including, amongst others, the uterine cervix, kidney, and brain (8).

This syndrome shows an autosomal dominant inheritance pattern with reduced penetrance, which likely decreases the rate of familial cases. In cases with PPB, about 80% of the *DICER1* germline pathogenic variants are inherited by a parent and nearly 20% are *de novo* (9).

This paper aims to review the clinical and genetic features of DICER1 syndrome, with particular focus on the description of the different types of cancer reported in this syndrome, grouped by systems.

## DICER1 Syndrome Genetics

The *DICER1* gene, located on chromosome 14q32.13, encodes an RNA endonuclease (Dicer) that is involved in the post-transcriptional gene expression of over 30% of protein-coding genes by modulating microRNAs (miRNAs) (10, 11).

miRNAs are transcribed as pri-miRNAs, that are longer precursor, which are elaborated into pre-miRNAs in the nucleus. The pre-miRNAs, transported to the cytoplasm, are processed by Dicer to give a ∼21-bp RNA duplex intermediate. One strand of this RNA is incorporated into the RNA-induced silencing complex (RISC), and matched to complementary mRNA targets to regulate gene expression, inhibiting mRNA degradation (12).

In most syndrome’s neoplasms a biallelic pathogenic variant in DICER1 has been detected: usually a germline loss-of-function pathogenic variant in one allele and a tumor-specific somatic hotspot variant in the second allele. Several studies have shown that “monoallelic *DICER1* inactivation promotes tumorigenesis, whereas biallelic loss is inhibitory, and although inactivation of one *DICER1* allele is the initiating event in DICER1 syndrome”, leading “to dysregulation of miRNA levels, other events must be required for cancer to occur ” (13, 14). Only one third of DICER1 carriers present a neoplasm during the life, hinting that multiple additional events are required (13, 14).

This process suggests a predominant haploinsufficient tumor-suppressor function, where one copy of Dicer, albeit mutated, is functioning, rather than a more classical “two-hit” tumor suppressor model, which has been described in association with earlier diagnosis of *DICER1*-related conditions, where no function of the oncosuppressor gene is preserved (5, 15, 16).

Complete loss of Dicer is incompatible with life (4, 17, 18), while somatic mosaic mutations in the RNase IIIb domain have been associated with a more serious form of DICER1 syndrome, named GLOW syndrome from Global developmental delay, Lung cysts, Overgrowth, and Wilms tumor (19). Functional evidence links the hotspot mutations in the RNAse IIIb domain to specific dysregulation of certain miRNAs leading to activation of the PI3K/AKT/mTOR pathway (20). This mechanistic link to the PI3K/AKT/mTOR pathway may explain the fact that GLOW syndrome shares some clinical features with other conditions characterized by somatic gain-of-function mutations of genes of this pathway, such as lung cysts, reported in Proteus syndrome, and segmental overgrowth, a prominent feature of PROS (21).

The recurrent involvement of specific organs (lungs, thyroid, kidneys, ovaries) in presence of *DICER1* alterations may lead to infer that the effects of miRNAs on gene expression are tissue-specific (19). Nonetheless, the penetrance of each of the *DICER1*-associated neoplasms in inherited conditions is not fully understood. Individuals carrying germline loss-of-function mutations may present clinical features in few sites (0–2) of their body, while patients with mosaic “hotspot” mutations are more prone to manifestations in multiple site (6).

## Clinical Features of Tumors Commonly Associated with *DICER1* Variants

**Figure 1:**
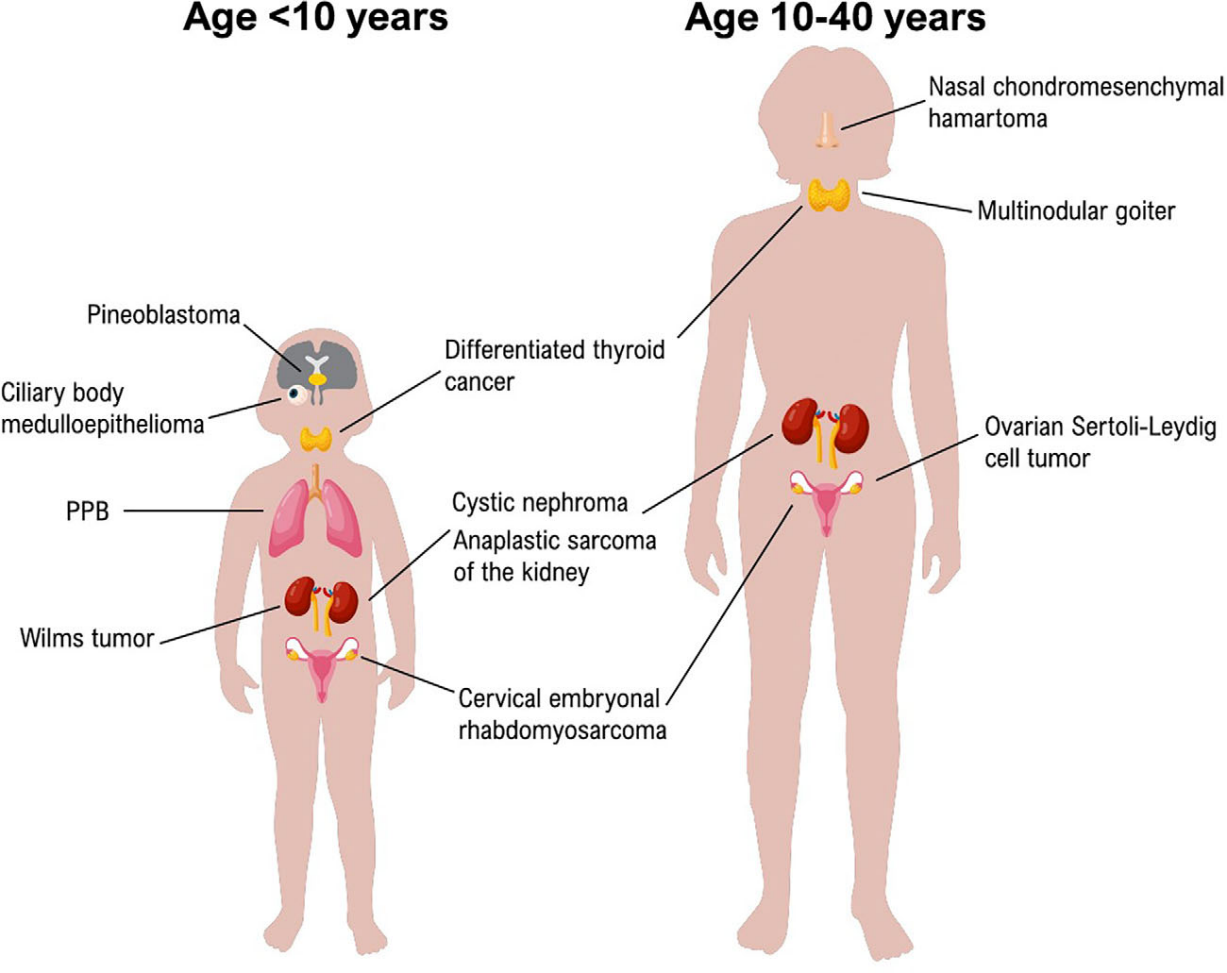
Principal DICER1-Syndrome neoplasms according to the age of onset.

Different tumors are related to *DICER1* syndrome as reported by Foulkes et al. and by Stewart et al. In **Figure 1** we resumed the principal neoplasms according to the age of onset.

Foulkes et al. in 2014 described the *DICER1*-associated features and their characteristics, as reported in **Table 1** (5).

**Table 1:** Key clinical phenotypes (ordered by relative frequency) associated with germline *DICER1* mutations.

Phenotype	Age (peak)
PBB	
Type I (cystic) PPB	0–24 m (8 m)
Type II (cystic/solid) PPB	12–60 m (31 m)
Type III (solid) PPB	18–72 m (44 m)
Type Ir (cystic) PPB	Any age
Multinodular goiter	5–40 y (10–20 y)
Cystic nephroma	0–48 m (undetermined)
Ovarian Sertoli-Leydig cell tumor	2–45 y (10–25 y)
Cervical embryonal rhabdomyosarcomas	4–45 y (10–20 y)
Differentiated thyroid cancer	5–40 y (10–20 y)
Wilms tumor*	3–13 y (undetermined)
Juvenile hamartomatous intestinal polyps*	0–4 y (undetermined
Ciliary body medulloepithelioma	3–10 y (undetermined)
Nasal chondromesenchymal hamartoma	6–18 y (undetermined)
Pituitary blastoma	0–24 m (undetermined)
Pineoblastoma	2–25 y (undetermined)
Anaplastic sarcoma of the kidney	Estimated 2–20 y
Medulloblastoma*	Undetermined
ERMS bladder*	Estimated <5 y
ERMS ovary	Undetermined
Neuroblastoma*	Estimated <5 y
Congenital phthisis bulbi*	Birth
Juvenile granulosa cell tumor*	Undetermined
Gynandroblastoma	Undetermined
Cervix primitive neuroectodermal tumor	Undetermined

*The association of these conditions with DICER1 variants may not be so strong to warrant testing in the absence of other features suggestive of DICER1 syndrome.

PBB, Pleuropulmonary blastoma; ERMS, embryonal rhabdomyosarcoma; m, months; y, years.

Stewart et al. recently published the first quantitative analysis of site-specific neoplasm risk, analyzing the standardized incidence ratios of 207 individuals carrying *DICER1* variants, selected combining data from three large cohorts of patients. The most remarkable rates were noted in PPB, in gynecologic tumors, especially SLCTs and rhabdomyosarcoma, and in cystic nephroma (1).

### Lung

#### Pleuropulmonary Blastoma

PPB is a rare tumor that develops during fetal life/infancy and constitutes the most common life-threatening manifestation of DICER1 syndrome (22). Type I PPB is typically a purely cystic mass occurring before age of 2 years, with a 5-year overall survival (OS) of 89% if it does not progress to type II or III PPB. Type II is a solid-cystic tumor while type III is purely solid; both types present from approximately 2 to 6 years of age and are malignant, although type III is generally more aggressive. If treated with chemotherapy and radiotherapy, OS rates may reach up to 74% in type II and 53% in type III. The fourth type, named type Ir, as “type I regressed”, is a cystic tumor lacking malignant cells and is supposed to represent regressed/non-progressed type I PPB. OS for this type of PPB is 100%. Cystic PPB is reported to be common in carriers of *DICER1* variants, and only a limited number of cases had a type II II or III PPB progression (1).

The PPB begins as a cystic lung lesion, also defined as a Type I PPB, a well-defined pathology entity with a potential evolution in a more aggressive tumor. We need to underline that the imaging findings of Type I PPB is overlapped with congenital lung cyst; congenital lung cyst with congenital pulmonary airway malformation (CPAM) are almost diagnosed in prenatal period or over the first year and a surgical approach—with pathology study—was mandatory only in symptomatic cases. Indeed, in more than 70% of CPAM, a wait and see strategy is addressed (23); in this cases a DICER1 variants should always be considered in order to identify promptly with a strict follow-up and genetic screening patients at risk of more aggressive PBB. The pathology should always consider PPB evaluating a CPAM.

Shortness of breath and pneumothorax due to cyst rupture may be the presenting symptoms of PPB.

### Thyroid

#### Multinodular Goiter and Epithelial Differentiated Thyroid Cancer

Multinodular goiter (MNG) is characterized by the development of thyroid nodular lesions. MNG is common in individuals with *DICER1* pathogenic variants, as reported by Khan et al. (24). Germline *DICER1* mutations have been reported in children with both MNG or familial MNG (25). The risk of DTC in carriers of *DICER1* variants is elevated as compared to the general population and its occurrence is typically related to an indolent course (26).

### Kidney

#### Cystic Nephroma, Wilms’ Tumor, and Anaplastic Sarcoma of the Kidney

Cystic nephroma is a benign multicystic kidney tumor that constitutes the most common neoplasm associated with PPB (3). It has a bimodal incidence: 65% of cases occur in the pediatric band, before the age of 4, while 35% of cases appear in adulthood and are usually seen between the fourth and the sixth decade (27, 28).

DICER1 syndrome also includes an elevated risk of Wilms’ tumor, an embryonal cancer of the kidney that affects children before the age of 6, without evidence to be a consequence of a prior cystic nephroma (29, 30).

Recent reports enumerate anaplastic sarcoma of the kidney in DICER1 syndrome, correlating the germline DICER1 mutations with the development of these tumors, and postulate that they may arise from pre-existing pediatric cystic nephromas (31–33).

### Gynecologic Manifestations

The gynecologic tumors most frequently associated to DICER1 syndrome are ovarian SLCTs and embryonal rhabdomyosarcoma of the cervix. These neoplasms, as well as PPB and MNG, constitute key features leading to consider an underlying cancer predisposition syndrome, especially if found in children or adolescents (34).

#### Ovarian Sertoli-Leydig Cell Tumor

Unlike PPB, the age range of increased risk for genital tract tumors is wide (2 to 40 years), even if some data suggest that ovarian SLCTs arising in patients carrying *DICER1* variants occur mostly in the second decade (18, 35). Moderately differentiated SLCTs are most common, but juvenile granulosa cell tumor (JGCT), gynandroblastoma, and unclassified sex cord-stromal tumors have also been described. Most tumors are stage I, presenting with androgenic symptoms and a pelvic mass, that rarely may be bilateral (34). The prognosis of ovarian SLCT is generally favorable, but a recent report indicates that somatic *DICER1* variants SLCTs may be linked to a higher relapse risk than others (36).

#### Cervical Embryonal Rhabdomyosarcomas

Even though rhabdomyosarcoma is the most common cervical sarcoma, it is still very rare (37). Most *DICER1* cases are confined to the cervix at diagnosis, presenting with polypoid appearances (botryoides) and with vaginal bleeding. Even if ERMS is one of the more common sarcomas in childhood, approximately a third of DICER1-related ERMS arises in patients older than 20 years (38, 39). Studies report a quite favorable prognosis with an EFS over 50%, and an OS around 90% (34, 38–41).

#### Other Ovarian Neoplasms

Poorly differentiated ovarian sarcoma (42), retiform SLCT, and primitive neuroectodermal tumor (PNET) of the cervix (43) have also been reported in individuals with possible germline *DICER1* variants.

### Central Nervous System

#### Pituitary Blastoma

Pituitary blastoma is an extremely rare tumor of the anterior pituitary. Genetic tests performed on 14 cases, on a total of 16 described to date, showed that all have at least one pathogenic variant in *DICER1* (44–46). For such reason, pituitary blastoma may be considered pathognomonic for DICER1 syndrome (46).

#### Pineoblastoma

Pineoblastoma is a rare primitive neuroectodermal grade IV tumor originating in the pineal gland (47). Only a few genes have been implicated in the pathogenesis of pineoblastomas, for instance, *RB1* in the setting of “trilateral retinoblastoma” (48).

To date, in *DICER1*-related pineoblastomas loss of heterozygosity of the wild-type *DICER1* allele seems to be the somatic event, in contrast from the typical missense hotspot mutations that usually lead to a factual germline heterozygosity (49–53). Moreover, somatic *DROSHA* and *DGCR8* mutations, both related to the Dicer miRNA-regulating pathway, have been recently documented in pineoblastomas, in addition to germline and somatic *DICER1* mutations (50), indicating that pineoblastoma development is influenced by disturbances of miRNA processes (46).

#### Others

Other brain tumors associated to DICER1 alterations have also been reported but their genetic association has not been clearly demonstrated. These include medulloblastoma (6, 54), intracranial medulloepithelioma (55), anaplastic meningeal sarcoma (53), glioblastoma multiforme (56, 57), and embryonal tumor with multilayered rosettes (ETMR) (58).

### Head and Neck

#### Ciliary Body Medulloepithelioma

Ciliary body medulloepithelioma is a rare embryonal ocular tumor, that arises from the eye’s ciliary body, which generally occurs during infancy and constitutes the second most common eye tumor of childhood, after retinoblastoma (59–61).

Some cases suspected to be *DICER1*-related have been documented but further studies are required to support their association with the syndrome (6, 15, 62–71).

#### Nasal Chondromesenchymal Hamartoma

Nasal chondromesenchymal hamartoma is a rare benign tumor of the sinus and nasal cavities that have been described in children with PPB. This peculiar association has led to the assumption that this hamartoma is also a manifestation of DICER1 syndrome (5, 72).

## Molecular Diagnostics

Molecular genetic testing methods, including single-gene or multigene panel testing, may be considered when clinical, imaging, and/or histopathological features evoke a DICER1 syndrome’s diagnosis. Heterozygosis is the most common condition through DICER1 syndrome’s patients, where commonly a germline loss‐of‐function gene variant (nonsense, frameshift, or splice-affected) generates a truncated protein. These variants can be identified by Sanger sequencing or next-generation sequencing (NGS). NGS has specific advantages over traditional Sanger sequencing, considered the gold standard for mutation analysis for many years, as multiple genes in several patients can be tested simultaneously. Indeed, when the phenotype is hard to distinguish from many other cancer predisposition syndromes, extensive genetic testing, based on multigene panels or exome analysis can be useful to identify the molecular defects underlying the condition.

Besides point mutations, other predisposing *DICER1* alterations have also been documented, including deletion of the entire *DICER1* locus (62), or intragenic deletions involving one or more exons (73). Methods used to detect these kinds of alterations may include quantitative polymerase chain reaction (PCR), multiplex ligation-dependent probe amplification (MLPA) and gene-targeted microarray. Finally, molecular genetic testing of tumor DNA may be necessary to identify somatic mosaicism, which is observed in 10% of individuals with DICER1 syndrome.

## Surveillance

Although risks of malignancy are elevated, most patients with pathogenic germline *DICER1* variants live healthy lives. Indeed, a tumor occurs in 19,3% of the patients who carry germline pathogenic variation by the age of 50 years old and the neoplastic risk rises with age, especially in females, that are exposed to the risk to present with gynecologic neoplasms (1).

Schultz et al. have defined the indications for *DICER1* genetic counseling and testing, and they also provided specific screening strategies to manage risk in carriers of *DICER1* pathogenic variants (2). Germline DICER1 genetic testing is to consider in individuals with one major or two minor criteria. “Major criteria are: PPB, lung cysts in childhood, thoracic embryonal rhabdomyosarcoma, cystic nephroma, genitourinary sarcomas including undifferentiated sarcoma, ovarian Sertoli−Leydig cell tumor, gynandroblastoma, uterine cervical or ovarian embryonal rhabdomyosarcoma, genitourinary/gynecologic neuroendocrine tumors, multinodular goiter or thyroid cancer in two or more first-degree relatives or in an index patient with a family history consistent with DICER1 syndrome, childhood-onset multinodular goiter or differentiated thyroid cancer, ciliary body medulloepithelioma, nasal chondromesenchymal hamartoma, pineoblastoma, pituitary blastoma. Minor criteria are: Lung cysts in adults, renal cysts, Wilms tumor, multinodular goiter or differentiated thyroid cancer, embryonal rhabdomyosarcoma other than thoracic or gynecologic, poorly differentiated neuroendocrine tumor, undifferentiated sarcoma, macrocephaly” (2).

Surveillance guidelines for individuals with a germline *DICER1* pathogenic variant have been established. The current guidelines include “chest radiograph every 4–6 months until age 8 years, and every 12 months until 12 years; a chest computed tomography scan should be considered. Baseline chest radiograph or chest CT should be considered when the diagnosis is performed after age 12 years. Thyroid ultrasound is recommended by the age of eight years with subsequent ultrasounds every three to five years. Individuals with a history of chemotherapy exposure should begin thyroid ultrasound within three to five years from treatment. Pelvic ultrasounds for surveillance for gynecologic tumors in females are recommended every 6 to 12 months by the age of eight years and extending until at least age 40 years. Screening for cystic nephroma and other renal tumors includes abdominal ultrasounds every six months until age eight years and then annually until age 12 years. Visual acuity measurement and dilated ophthalmology examination for ciliary body medulloepithelioma is recommended annually from age three years until at least age ten years. Annual physical examination should be considered by an expert clinician” (2).

## Therapeutic Perspectives

Some studies explored the use of metformin to upregulate DICER1 and linked proteins in mice, to counter the DICER1 syndrome’s effects (74–77). Despite patients affected by biallelic DICER1 mutations may not benefit from this treatment, metformin will be may proposed to patients with a single allele alteration, to try to augment DICER1 protein production and compensate the deficit, preventing the oncogenetic cascade.

## Conclusions

DICER1 syndrome is a rare condition caused by germline variants of *DICER1*; the occurrence of a second somatic tissue-specific mutation leads to different phenotypes ranging from benign lesions to malignant tumors. Screening for *DICER1* variants should be performed in all patients with PPB and considered in few benign lesions and malignant tumors. A prompt identification of this syndrome is necessary to plan a correct follow-up and screening for tumor occurrence during the patient’s lifetime.

## Author Contributions

AC reviewed the literature and was a major contributor in writing the manuscript. MI reviewed the literature and wrote the manuscript. LB contributed to the concept and reviewed critically the manuscript. AM contributed to the concept, reviewed the literature, and reviewed critically the manuscript. All the authors state that no honorarium, grant, or other form of payment was given to anyone to produce the manuscript. All authors contributed to the article and approved the submitted version.

## Conflict of Interest

The authors declare that the research was conducted in the absence of any commercial or financial relationships that could be construed as a potential conflict of interest.
